# Optogenetic Evidence for a Direct Circuit Linking Nociceptive Transmission through the Parabrachial Complex with Pain-Modulating Neurons of the Rostral Ventromedial Medulla (RVM)

**DOI:** 10.1523/ENEURO.0202-17.2017

**Published:** 2017-06-26

**Authors:** QiLiang Chen, Zachary Roeder, Ming-Hua Li, YangMiao Zhang, Susan L. Ingram, Mary M. Heinricher

**Affiliations:** 1 Department of Neurological Surgery, Oregon Health & Science University, Portland, OR 97239; 2 Department of Behavioral Neuroscience, Oregon Health & Science University, Portland, OR 97239; 3 Neuroscience Graduate Program, Oregon Health & Science University, Portland, OR 97239

**Keywords:** brainstem, descending control, pain modulation, raphe, rat

## Abstract

The parabrachial complex (PB) is a functionally and anatomically complex structure involved in a range of homeostatic and sensory functions, including nociceptive transmission. There is also evidence that PB can engage descending pain-modulating systems, the best characterized of which is the rostral ventromedial medulla (RVM). Two distinct classes of RVM neurons, “ON-cells” and “OFF-cells,” exert net pronociceptive and anti-nociceptive effects, respectively. PB was recently shown to be a relay of nociceptive information to RVM ON- and OFF-cells. The present experiments used optogenetic methods in a lightly anesthetized rat and an adult RVM slice to determine whether there are direct, functionally relevant inputs to RVM pain-modulating neurons from PB. Whole-cell patch-clamp recordings demonstrated that PB conveys direct glutamatergic and GABAergic inputs to RVM neurons. Consistent with this, *in vivo* recording showed that nociceptive-evoked responses of ON- and OFF-cells were suppressed by optogenetic inactivation of archaerhodopsin (ArchT)-expressing PB terminals in RVM, demonstrating that a net inhibitory input to OFF-cells and net excitatory input to ON-cells are engaged by acute noxious stimulation. Further, the majority of ON- and OFF-cells responded to optogenetic activation of channelrhodopsin (ChR2)-expressing terminals in the RVM, confirming a direct PB influence on RVM pain-modulating neurons. These data show that a direct connection from the PB to the RVM conveys nociceptive information to the pain-modulating neurons of RVM under basal conditions. They also reveal additional inputs from PB with the capacity to activate both classes of RVM pain-modulating neurons and the potential to be recruited under different physiological and pathophysiological conditions.

## Significance Statement

Pain-modulatory circuits receive input from ascending pain transmission pathways as part of a recurrent network. Using optogenetics with whole-cell patch-clamp recording and *in vivo* single-cell recording, the present studies identified direct functional connections from the parabrachial complex (PB), a major target of ascending nociceptive pathways, to physiologically identified pain-modulating neurons of the rostral ventromedial medulla (RVM), the primary output node of a major descending pain-modulating system. These data for the first time point to an identified nociceptive synapse in RVM that could be probed in relevant physiologic contexts, and set the stage for a dissection of the links between nociceptive transmission and nociceptive modulation in the transition from acute to chronic pain.

## Introduction

Descending pain-modulatory circuits mediate top-downregulation of nociceptive processing, transmitting cortical and limbic influences to the dorsal horn. These modulatory pathways are also intimately intertwined with ascending transmission pathways as part of positive and negative feedback loops. However, circuits through which ascending nociceptive information gains access to descending pain-modulatory systems are only now being defined.

The parabrachial complex (PB) is a functionally and anatomically complex structure involved in a range of homeostatic and sensory functions (Sakai and Yamamoto, 1998; Morrison, 2011; Kaur et al., 2013; Davern, 2014; Han et al., 2015; Yokota et al., 2015; Meek et al., 2016; Roman et al., 2016; Sammons et al., 2016), including nociception (Gauriau and Bernard, 2002; Neugebauer, 2015). PB receives nociceptive input via the spinoparabrachial tract. Nociceptive neurons have been identified in the PB, with the highest density in the lateral region (Bernard et al., 1994; Hermanson and Blomqvist, 1996; Bourgeais et al., 2001). A subset of nociceptive PB neurons have been implicated in recruitment of amygdala circuits important for the affective dimension of pain (Neugebauer, 2015). However, in addition to this well-documented role as part of an ascending nociceptive pathway, PB can engage descending pain-modulating systems (Lapirot et al., 2009; Roeder et al., 2016), which in turn project back to the dorsal horn to influence nociceptive processing.

The best-characterized brainstem pain-modulating system includes links in the midbrain periaqueductal gray and rostral ventromedial medulla (RVM; Heinricher et al., 2009; Heinricher and Fields, 2013). The RVM can facilitate or suppress nociceptive transmission at the level of the dorsal horn through the actions of two distinct classes of neurons, “ON-cells” and “OFF-cells,” which respectively exert pronociceptive and anti-nociceptive effects. Both classes receive noxious inputs: ON-cells are activated, leading to a “burst” of activity associated with behavioral responses to noxious stimulation, while OFF-cell firing is suppressed, producing a “pause” in any ongoing activity. Although these reflex-related changes in ON- and OFF-cell firing are critical to their pain-modulating function (Fields and Heinricher, 1985; Heinricher et al., 2010), the pathways through which nociceptive information is conveyed to the RVM have only recently begun to be delineated, with PB identified as one important relay (Roeder et al., 2016).

Because of the structural and functional complexity of PB efferent projections, defining the pathways through which PB exerts its influence on RVM pain-modulating neurons is challenging. Although PB can be shown to project directly to RVM using bulk tracer methods (Beitz, 1982; Verner et al., 2008), whether that projection has a role in pain modulation or in one of the other functions shared by these two regions is unclear. Moreover, PB has abundant projections to other structures that are themselves implicated in pain modulation and project directly to the RVM, including the periaqueductal gray, insula, and amygdala (McGaraughty and Heinricher, 2002; Jasmin et al., 2003; McGaraughty et al., 2004; Sato et al., 2013).

A direct connection from PB to the RVM pain-modulating neurons would allow PB to contribute to positive and negative intrabrainstem feedback loops, promoting or limiting development and maintenance of pathologic pain states (Porreca et al., 2001; Ren and Dubner, 2002; Heinricher et al., 2009; De Felice et al., 2011). The present experiments used optogenetic methods in both the adult RVM slice and intact, lightly anesthetized rats to test the hypothesis that there are direct, functionally relevant PB inputs to RVM pain-modulating neurons, and that the two classes of pain-modulating neurons, ON- and OFF-cells, receive distinct influences from PB. We used a channelrhodopsin (ChR2)-assisted circuit-mapping approach in an RVM slice to determine direct synaptic inputs to RVM. We then used ChR2 *in vivo* to determine the net effect of PB-terminal stimulation on identified RVM pain-modulating neurons. While activation of PB terminals using ChR2 provided evidence that inputs exist and have the potential to be activated, this approach does not imply that a given input is in fact activated under any given condition. Complementary *in vivo* studies therefore used archaerhodopsin (ArchT) expressed in PB terminals to determine what PB inputs to RVM are recruited by acute noxious stimuli. Our data indicate that the PB projection to the RVM constitutes a direct connection that conveys nociceptive information to this pain-modulating circuit.

## Materials and Methods

All experimental procedures were approved by the Institutional Animal Care and Use Committee at Oregon Health & Science University and followed the guidelines of the Committee for Research and Ethical Issues of the International Association for the Study of Pain.

### Vector injection

**Figure 1.**
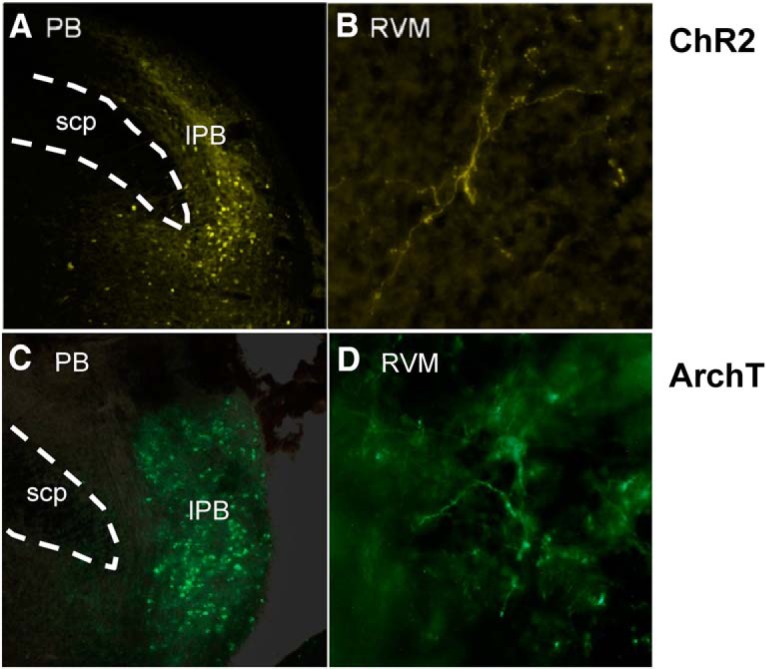
Examples of ChR2 and ArchT expression in the lateral PB region and terminal expression in RVM. Examples of ChR2 expression in (***A***) PB cell bodies and (***B***) PB projecting fibers in RVM. Examples of ArchT expression (***C***) PB cell bodies and (***D***) PB projecting fibers in RVM. lPB, lateral PB region; scp, superior cerebellar peduncle.

**Figure 2.**
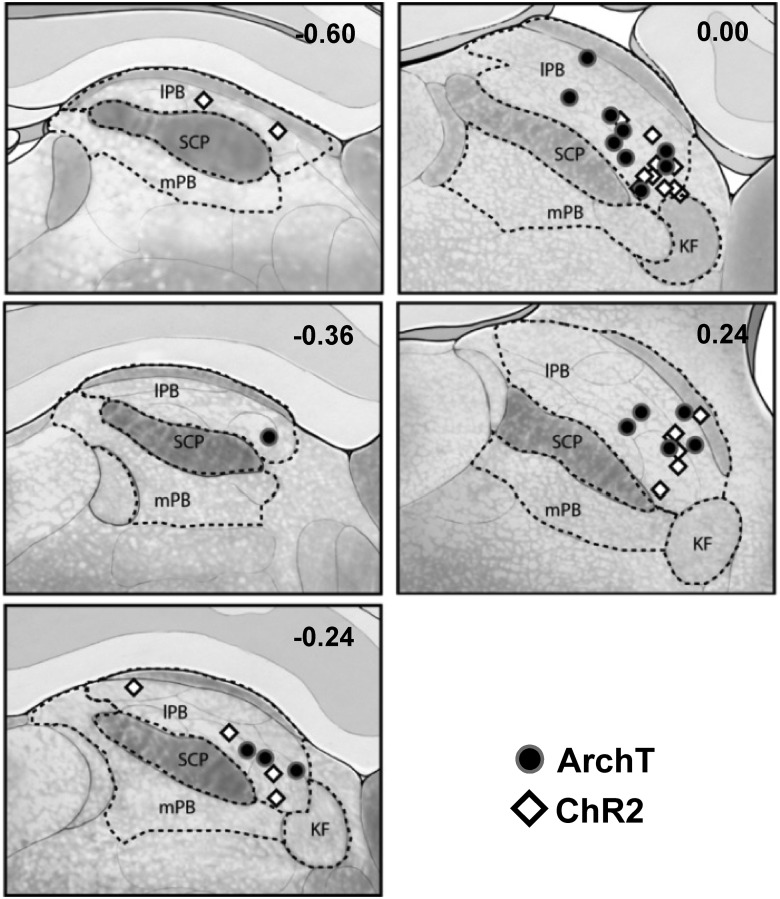
Vector injection sites in PB. Injection sites were distributed among sections at +0.24 to −0.36 mm relative to the interaural line. Injections inside the lateral PB (lPB) area were considered on-target. KF, Kölliker-Fuse; mPB, medial PB area; SCP, superior cerebellar peduncle.

Rats (90–110 g, male Sprague Dawley, Charles River Laboratories) were deeply anesthetized using isoflurane. The rat was placed in a stereotaxic apparatus, and maintained on a circulating warm-water pad. A craniotomy was performed ∼1-3 mm lateral to the sagittal suture and 0.5–2.5 mm rostral to the lambdoid suture, and the dura removed to allow the placement of a microinjection cannula with a 14° angle to the coronal plane into the lateral parabrachial nucleus (anterior-posterior: −1.2 mm, medial-lateral: ± 2.3 mm, medial-lateral: +3 mm from interaural). AAV9-hSyn-ChR2-eYFP or AAV9-CAG-ArchT-eGFP (200 nl, Penn Vector Core, University of Pennsylvania) was microinjected into the lateral PB, since lateral, but not medial, PB relays nociceptive information to the RVM (Roeder et al., 2016). The microinjection cannula was left at the injection site for 10 min before retraction to minimize back-flow. The craniotomy site was filled with gelfoam, and the incision site closed. Rats received lidocaine ointment, penicillin G (80 kU/ml, i.m.) and carprofen (5 mg/ml, s.c., Pfizer) after the procedure, and were returned to the home cage for 2–5 weeks to allow expression of ChR2 and ArchT. Examples showing reporter in cell bodies in PB and in terminals in RVM are shown in Figure 1. A plot of ChR2 and ArchT injection sites in PB is provided in Figure 2 (anatomic control sites surrounding PB were not tested because previous work has shown that lateral PB is the relevant source of nociceptive inputs to the RVM; Roeder et al., 2016).

### *In vitro* whole-cell patch-clamp recordings

#### RVM slice preparation

The RVM slice was prepared as described previously (Li et al., 2015). Two to five weeks after vector injection, rats were deeply anesthetized with isoflurane, and the brains rapidly removed and placed in n-methyl-D-glucamine (NMDG)-sucrose based “cutting buffer” containing 52 mM NMDG, 2.5 mM KCl, 0.5 mM CaCl_2_, 10 mM MgSO_4_, 1.2 mM NaH_2_PO_4_, 30 mM NaHCO_3_, 25 mM D-dextrose, 75 mM sucrose, 5 mM sodium ascorbate, 2 mM thiourea, and 3 mM sodium pyruvate, at pH 7.4 (adjusted with HCl) and 300–310 mOsm (Zhao et al., 2011; Ting et al., 2014). Coronal slices (180–200 µm) were cut in 95% O_2_/5% CO_2_ cutting buffer. Slices were incubated at 35°C in a submerged chamber containing artificial cerebrospinal fluid (aCSF) equilibrated with 95% O_2_/5% CO_2_ for at least 30 min and subsequently maintained at room temperature until transfer to a recording chamber held at 30–31°C. The aCSF contained 126 mM NaCl, 2.5 mM KCl, 2.4 mM CaCl_2_, 1.2 mM MgCl_2_, 1.2 mM NaH_2_PO_4_, 21.4 mM NaHCO_3_, and 11.1 mM D-dextrose, pH 7.4, and 300–310 mOsm.

#### Whole-cell patch-clamp recordings

Voltage-clamp recordings (holding potential −70 mV) were made from RVM neurons in the whole-cell configuration using an Axopatch 200B amplifier (Molecular Devices). Patch clamp electrodes were pulled from borosilicate glass (1.5 mm in diameter; WPI) on a two-stage puller (PP83, Narishige). Pipettes had a resistance of 2–4 MΩ. Postsynaptic currents were recorded in an intracellular pipette solution containing 140 mM CsCl, 10 mM HEPES, 4 mM MgATP, 3 mM NaGTP, 1 mM EGTA, 1 mM MgCl_2_, and 0.3 mM CaCl_2_, pH adjusted to 7.3 with CsOH, 290 mOsm. Series resistance (<20 MΩ) was compensated by 70–80% and continuously monitored during experiments. A junction potential of −5 mV was corrected during recording. GABAergic events were isolated in the presence of glutamate receptor antagonists NBQX (10 µM) and kynurenic acid (KA; 100 µM). Glutamatergic events were isolated in the presence of the GABA_A_ receptor antagonist bicuculline (BIC; 30 µM). Recordings in which access resistance or capacitance changed by >15% during the experiment were excluded from data analysis. Latency of synaptic currents was measured as latency at 5% of the peak current and jitter as the SD of latency.

### In vivo recording

Four to five weeks after vector injection in the PB, animals were deeply anesthetized (isoflurane) and a catheter inserted in the external jugular vein for subsequent infusion of the short-acting barbiturate methohexital. They were then transferred to a stereotactic frame. Small craniotomies were made to gain access to the RVM and PB, and the meninges were opened. Heart rate was monitored using EKG. Body temperature was monitored and maintained at 36–37°C with a heating pad.

When preparatory surgeries were complete, rats were placed on a continuous methohexital infusion. The anesthetic plane was set at a depth that allowed a stable heat-evoked hindpaw withdrawal reflex, while preventing spontaneous movement.

Extracellular single-unit recordings were made using an optoelectrode, constructed by pairing a stainless-steel microelectrode (Microprobes) with gold- and platinum-plated tips with an optical fiber (200 µm in diameter, ThorLabs). Signals were amplified (10 k) and bandpass-filtered (400 Hz to 15 kHz) before analog-to-digital conversion at 32-k samples/s.

### Validating opsin functions

A subset of ChR2- or ArchT-injected animals was used to confirm that ChR2 expression allows light-induced activation of PB neurons, and that ArchT expression results in light-evoked inhibition of PB neurons. A neuron in lateral PB was isolated, and classified as nociceptive based on increased firing rate during a noxious heat stimulus applied to the plantar surface of the hindpaw using a Peltier device (Yale Instrumentation Service). A light source with mean wavelength of 470 nm (range: 465–475 nm, 1.1 mW power) was used to activate ChR2. A source with mean wavelength of 565 nm (range: 540–575 nm, 0.5 mW maximum power) was used to activate ArchT (both from Thorlabs).

### Recording RVM neurons in response to PB terminal manipulations

An RVM neuron was isolated and classified as an ON-, OFF-, or NEUTRAL-cell (Fields and Heinricher, 1985; Barbaro et al., 1986) using a noxious heat stimulus applied to the plantar surface of the hindpaw (Peltier device as above). The surface temperature was held at 35°C between trials, and then increased at a rate of 1.2°C/s from 35°C to a maximum of 53°C. Withdrawals were recorded as hamstring muscle electromyographic response (EMG), with the first positive inflection of the rectified EMG used as the onset of the response. ON-cells exhibit a burst of action potentials beginning immediately before a nocifensive withdrawal, or continue firing if already active. OFF-cells cease firing (if active), or remain silent (if inactive). To further confirm cell classification, a 10 s noxious pinch was delivered to the hindpaw using a toothed forceps: ON-cells fire and OFF-cells cease firing throughout the stimulus. All remaining neurons (i.e., those not showing a withdrawal-related burst or pause) were classified as NEUTRAL-cells. To avoid misclassifying any ON-cells with continuous ongoing activity as NEUTRAL-cells (Barbaro et al., 1986), a bolus of methohexital anesthetic was given to the point that the withdrawal reflex was abolished. Firing of NEUTRAL-cells is unaltered during this maneuver, whereas spontaneously active ON-cells slow or stop. After confirmation of cell type, animals were restabilized at a constant anesthetic flow rate before beginning data collection.

For the ArchT experiments, we recorded RVM responses to optogenetic manipulations of cell bodies in PB and PB terminals in RVM. Three trials with light-induced inhibition of PB terminals in RVM were performed, followed by three additional trials with light-induced inhibition of cell bodies in PB. Light trials (terminal or cell body stimulation) consisted of ∼60 s of continuous light stimulation, with a heat stimulus delivered in the second half of the light period, and ∼5 min between trials. No-light/heat-only trials were performed before every light stimulation to provide a control heat-related response. Because the densest spinoparabrachial projection ascends to the contralateral PB (Bernard et al., 1995; Feil and Herbert, 1995; Bester et al., 2000; Todd et al., 2000; Polgár et al., 2010), the heat stimulus was delivered to the paw contralateral to PB opsin expression.

For the ChR2 experiments, only terminal stimulations were performed. Each light trial consisted of ∼45 s of total light stimulation, with a heat trial in the last 30 s of the light train. To screen RVM neuron responses to pulses of different widths and frequencies, six different stimulation protocols were used for all isolated RVM neurons: continuous light, 50 ms at 10 Hz, 20 ms at 10 Hz, 10 ms at 10 Hz, 20 ms at 20 Hz, and 20 ms at 40 Hz, delivered in that order, with 2–5 min between stimulus trains. Each light protocol was tested once. To control for possible antidromic activation of PB cell bodies by terminal stimulation, 4% lidocaine (200 nl) was microinjected in PB in some experiments. Following the injection, two additional sets of terminal stimulation were performed in these animals (continuous light and 50 ms pulses at 10 Hz).

### Histology

**Figure 3.**
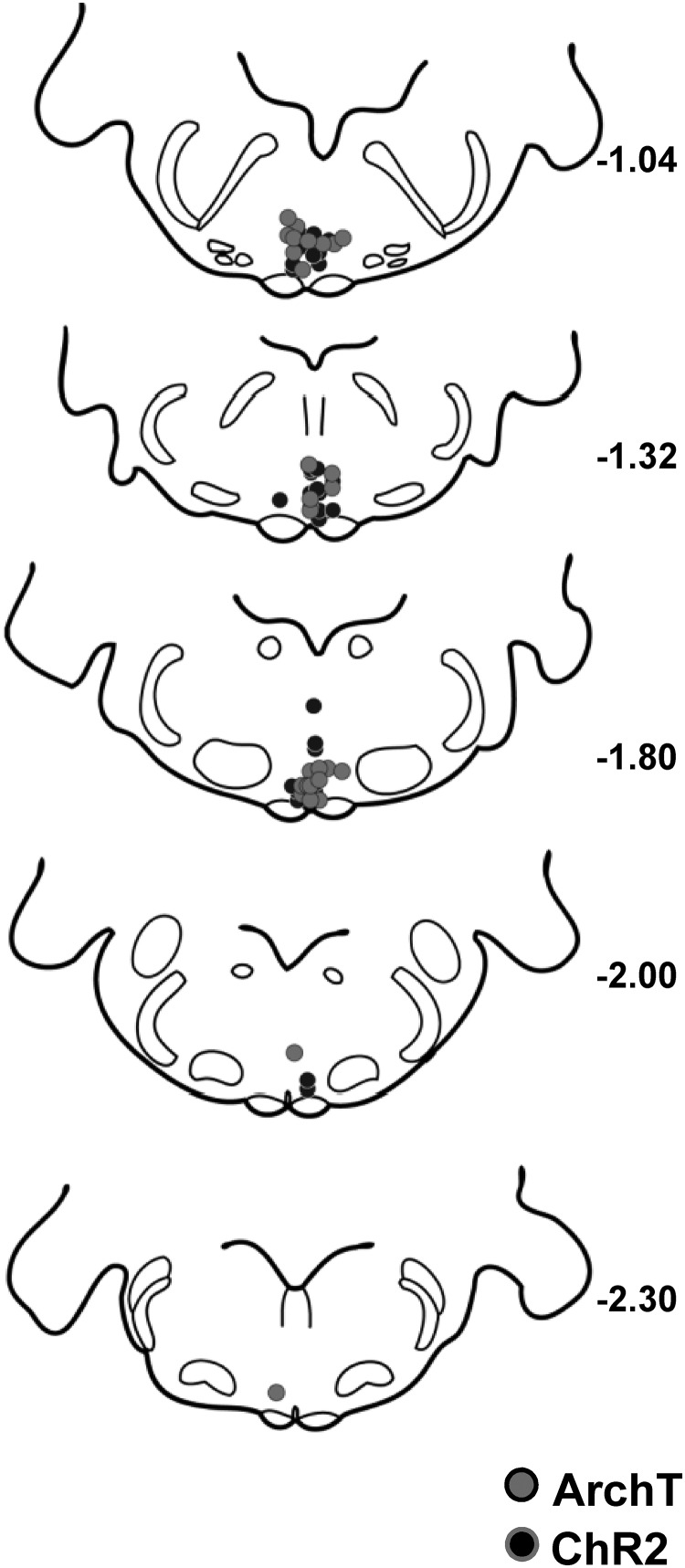
Recording sites within the RVM. Recording sites for both ArchT (gray circles) and ChR2 (black circles) experiments were distributed between −1.04 and −2.30 mm (relative to the interaural line). The majority of cells were isolated between −1.04 and −1.80 mm.

At the completion of the electrophysiological experiment, recording sites were marked with an electrolytic lesion (Fig. 3). Animals were then overdosed with methohexital, and perfused transcardially with saline followed by 10% formalin. The brains were removed, blocked, and sectioned (40 μm). Recording sites as well as viral expression in PB cell bodies and in terminals around the recording sites in the RVM were plotted on hand-drawn sections in Adobe Illustrator using landmarks defined by Paxinos and Watson (2009). Only data from “on-target” reporter expression and recording sites were analyzed. The RVM was defined as the nucleus raphe magnus and adjacent reticular formation at the level of the facial nucleus (−1.32 to −3.12 mm from interaural line, ±0.6 mm lateral, and 9–10 mm ventral to the brain surface).

### Analysis

The microelectrode signal, EMG, and EKG output were digitized and collected using Spike2 software (Cambridge Electronics Design). Each wave form was sorted using Spike2 template matching and cluster analysis, verified on an individual spike basis.

We characterized reflex-related changes in OFF-cell activity by the duration of the reflex-related pause. ON-cell activity was quantified as the total number of spikes in each reflex-related burst (which may last for many seconds or even minutes). In ChR2 experiments in which a reflex-related burst could not be defined due to light-evoked activation, the number of spikes in the 3-s period beginning 0.5 s before the paw withdrawal reflex was used in place of total evoked spikes.

Spontaneous activity was defined as the firing rate in the 10-s period before each stimulus (heat or light) or stimulus train (light). To assess the effect of each light-stimulation protocol, a ratio of firing during the light stimulus to that before stimulus onset was obtained. A change of >50% was considered an effect. If the cell was silent before stimulus onset, an increase of <10 spikes was considered no difference.

We also measured the latency of the cell response to light pulses in ChR2 experiments. To calculate the response latency for those cells with excitatory inputs, a peri-stimulus time histogram (0.5-ms bins) was constructed. The mean and SD of the control firing rate was calculated for a 10-s period before light onset, and the trial-specific response latency defined as the time at which the light-induced firing rate exceeded a threshold of 1.95 SDs above the prestimulation mean. The average response latency over all light-pulse trials was calculated for each light-evoked response. To calculate response latency for cells with inhibitory responses to optogenetic activation of PB afferents, interspike intervals were used.

EMG was rectified and smoothed, and area under the curve (AUC) for 15 s beginning at response onset (or cutoff in two cases with no response) calculated. A prestimulus 15-s “baseline” was subtracted for each trial. AUC during terminal or cell body inactivation was expressed as a percentage of that during no-light trials, reflecting the impact of ArchT inhibition of PB terminals or cell bodies on the magnitude of paw withdrawal.

Parameters describing ON- and OFF-cell firing (spontaneous firing rate, pause duration, burst) are typically highly right-skewed, and were log-transformed and analyzed using paired *t* test, or one-way ANOVA with repeated measures and *post hoc* Dunn’s multiple comparison test. Light versus prelight ratios were analyzed using Wilcoxon’s signed rank tests. These cell parameters are presented as geometric mean ± 95% confidence intervals. Other data are reported as mean ± SEM. Paw withdrawal latencies were analyzed using nonparametric tests (Wilcoxon’s signed rank test, Friedman’s test) because some responses were at the cutoff value. EMG magnitude was normalized to no-light trials for each cell, and compared with 1.0 using a one-sample *t* test. For all tests, *p* < 0.05 was considered significant.

## Results

### Expression of ArchT or ChR2 on neurons and terminals projecting from lateral PB to RVM

ArchT or ChR2 viral vectors were injected in the right PB two to five weeks before the electrophysiological experiment. Reporter proteins were expressed along the fibers and terminals projecting from PB. Positive fibers were found in regions consistent with a previous study using anterograde tracing methods (Saper and Loewy, 1980), and with other studies employing retrograde tracers (Beitz, 1982; Verner et al., 2008; Roeder et al., 2016). In the current study, PB fibers were seen to project throughout the RVM (raphe magnus nucleus, raphe pallidus nucleus, gigantocellular reticular nucleus), spanning the length of the RVM from the facial nerve (−1.32 mm from interaural line) to the caudal end of the facial nucleus (−3.12 mm from interaural line). In addition to the RVM, reporter-expressing fibers of similar density were also found in rostroventrolateral reticular nucleus and the facial nucleus. There were also lower-density positive fibers in other reticular nuclei, the solitary nucleus, abducens nucleus, and in the dorsal part of medial longitudinal fasciculus.

### Validation of light-evoked changes in firing in animals expressing ChR2 and ArchT in PB neurons

**Figure 4.**
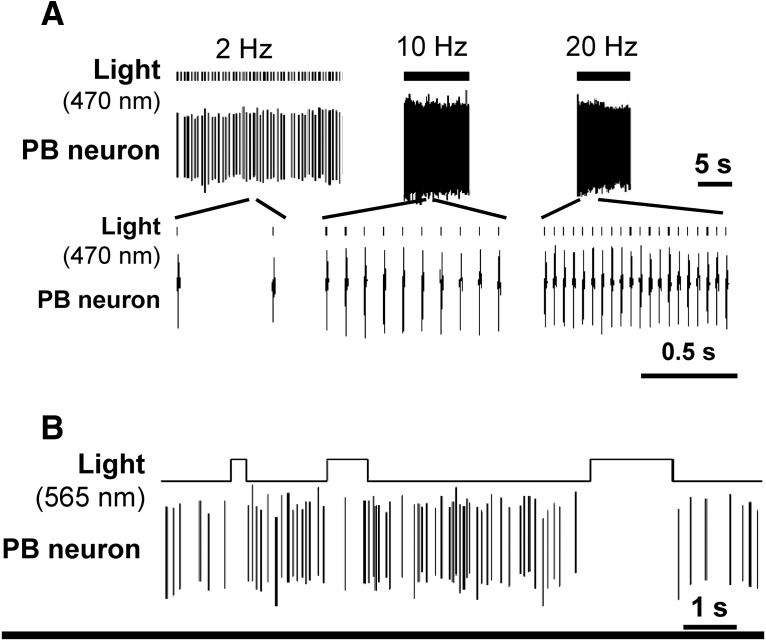
Validation of ChR2 and ArchT function on PB neurons. ***A***, Activation of PB neuron by light-induced activation of ChR2 (1- to 2-ms pulses at 2, 10, or 20 Hz). Firing of the neuron reliably followed the light trains. ***B***, Suppression of activity of a PB neuron during light-induced activation of ArchT.

A subset of ChR2- or ArchT-injected animals was used to ensure that ChR2 expression supported light-induced activation of PB neurons and that ArchT expression resulted in light-evoked inhibition of PB neurons. Most PB neurons sampled exhibited alternating silent and active phases in the absence of photostimulation, similar to those seen in RVM neurons (Barbaro et al., 1989). An example of light-induced activation of a PB neuron expressing ChR2 is shown in Figure 4*A*. Light trains were administered (1- to 2-ms pulses at 2, 10, or 20 Hz) during inactive phases. As illustrated in the representative trace, this PB neuron fired one spike with each light pulse at all three frequencies tested. An example of light-induced inhibition of a PB neuron expressing ArchT is shown in Figure 4*B*. Three light pulses of different durations were applied during an active phase of the recorded neuron. With activation of ArchT, firing of this neuron was noticeably depressed and even ceased entirely.

### Direct PB projections release either glutamate or GABA onto single RVM neurons

**Figure 5.**
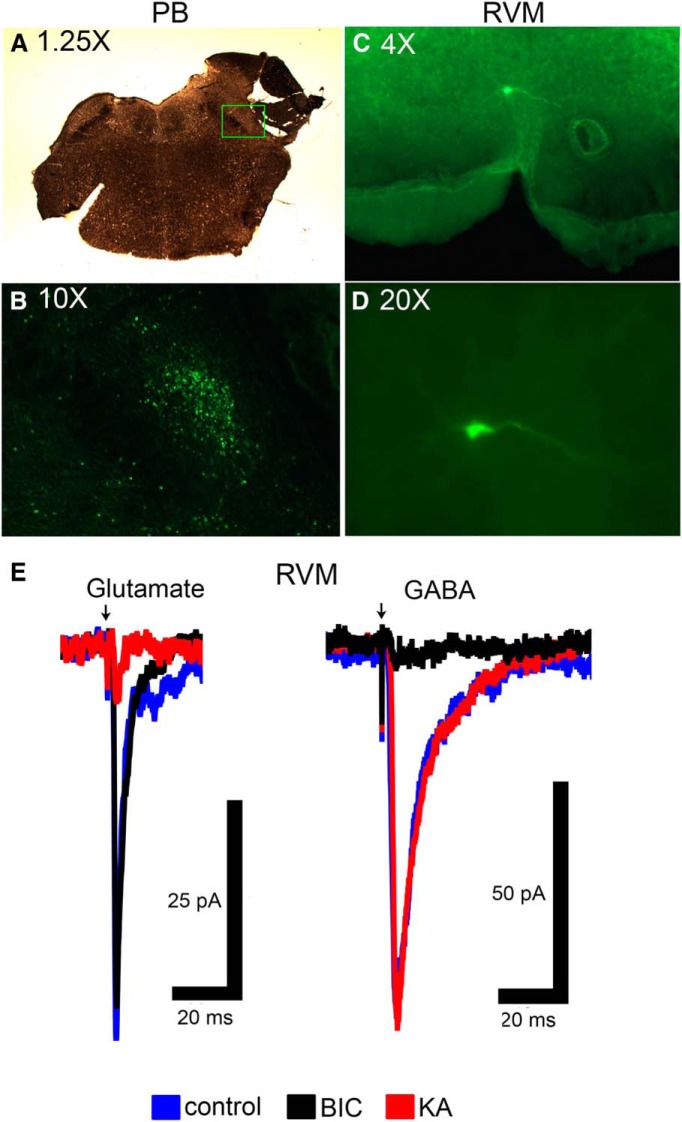
In vitro electrophysiology shows evidence for direct, functional PB inputs to individual RVM neurons. ***A***, Micrograph of slice showing area of PB microinjection. ***B***, Expression of reporter for ChR2 construct in PB in area outlined by square in ***A***. ***C***, Low magnitude (4×) picture of RVM slice containing a biocytin-labeled neuron that was filled during recording. ***D***, High magnification (20×) of biocytin-labeled cell in ***C***. ***E***, Light-evoked stimulation elicited either glutamatergic synaptic currents that were inhibited by KA but not BIC, or GABAergic synaptic currents that were inhibited by BIC but not KA. Note the difference in synaptic decay kinetics. Arrow indicates delivery of light stimulus.

To determine if there are direct synaptic connections from PB to individual RVM neurons, we used whole-cell patch-clamp methods in acute RVM slices taken from adult animals. An example of opsin expression in PB and the location of a recorded RVM neuron are shown in Figure 5*A–D*. Light stimulation of terminals expressing ChR2 in RVM slices elicited synaptic currents in 29 neurons (Fig. 5*E*). Of those 29, 21 had synaptic currents that were blocked by the glutamate antagonists KA or NBQX but were unaffected by the GABA_A_ receptor antagonist BIC, indicating a direct glutamatergic projection (Fig. 5*E*, left). The latency of onset of synaptic currents was 2.3 ± 0.2 ms with jitter (STD of latency) of 408 ± 26 µs (*n* = 21). The remaining 8/29 RVM neurons had light-evoked synaptic currents that were blocked by BIC but were unaffected by glutamate antagonists, indicating that they were direct GABAergic inputs (Fig. 5*E*, right). The GABAergic synaptic currents had longer decay kinetics and longer latency of onset (3.8 ± 0.6 ms; *t*_6.9_ = 2.53 with Welch’s correction, *p* = 0.039). The observed jitter was 622 ± 112 µs and was not significantly different from the glutamatergic synaptic currents (*t*_6.7_ = 1.871 with Welch’s correction, *p* = 0.106). Both synaptic currents occurred within the expected timeframe for monosynaptic connections. These data provide evidence for direct PB inputs to RVM neurons, and suggest that these inputs release either glutamate or GABA, but not both, onto individual RVM neurons.

### RVM neurons can be activated or inhibited by optogenetic activation of PB terminals in RVM in ChR2-injected animals in vivo

The *in vitro* studies documented direct glutamatergic and GABAergic synaptic connections to RVM neurons from PB. However, the recorded neurons were not functionally identified as ON-cells, OFF-cells, or NEUTRAL-cells in these slice studies. In a second set of experiments, we tested whether direct inputs from PB to RVM could alter activity of neurons in these three different classes. Using rats in which ChR2 viral vectors were injected in PB, we examined the net effect of stimulating PB terminals in RVM. Responses of 13 ON-cells, 14 OFF-cells, and 9 NEUTRAL-cells to light-induced activation of PB terminals in RVM were recorded in 28 animals. A range of light pulse widths and frequencies were tested (see Materials and Methods). We also determined whether optogenetic activation of PB terminals in RVM altered heat-evoked neuronal and behavioral responses. Unlike in the slice recording experiments, responses recorded *in vivo* reflects influences mediated by local circuits as well as any direct inputs to the cell being recorded.

**Figure 6.**
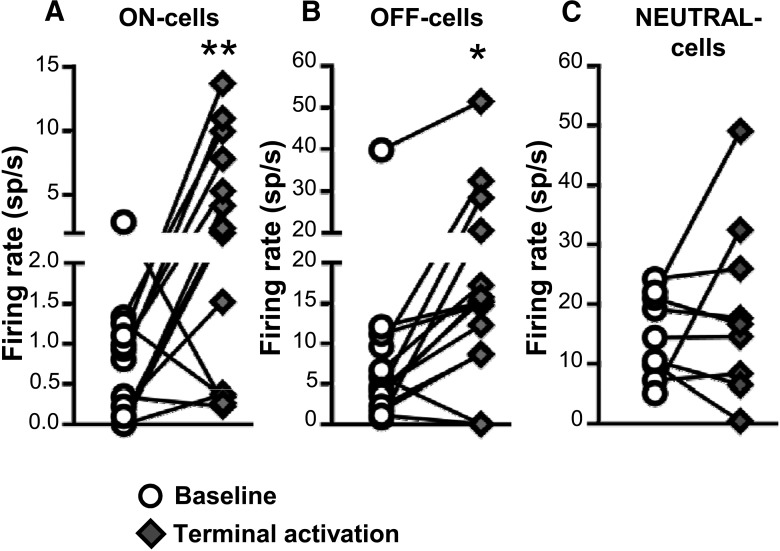
Effect of ChR2-induced activation of PB terminals on ongoing firing of ON-, OFF-, and NEUTRAL-cells in RVM. Terminal activation significantly increased the net firing rate of both ON- and OFF-cells (***A***, ***B***) but had no net effect on NEUTRAL cells (***C***). *p < 0.05 and **p < 0.01 compared with baseline, paired *t* test, *n* = 9–14 cells per class. Effects of different stimulation parameters are pooled for this analysis.

Both ON- and OFF-cells responded to light-induced activation of PB terminals in RVM, with overall increases in the firing of both classes (Fig. 6*A*,*B*). As a class, NEUTRAL-cells did not show a significant overall increase in firing during light-induced activation of PB terminals (Fig. 6*C*). For ON-cells, 8 of 13 cells recorded (62%) showed an increase in firing rate during the light pulse train, one cell exhibited a decrease in firing rate, and 4 cells showed no change in activity. As a whole, the population exhibited a 3.4 ± 1.1 spikes/s increase in overall firing rate with these stimulation protocols. Similarly, 10 of 14 OFF-cells (71%) displayed increased firing rate in response to light, two cells showed a decrease in spontaneous firing rate, and two cells showed no response. As a whole, OFF-cell firing was increased by 8.0 ± 2.0 spikes/s. Most NEUTRAL-cells were unresponsive, with two of nine NEUTRAL-cells recorded demonstrating an increase in overall firing, and one a decrease. The remaining six cells (67%) exhibited no change in firing. The mean change in NEUTRAL-cell firing was 4.2 ± 4.5 spikes/s.

The varied responses to PB terminal activation were not due to recruitment of other non-RVM PB targets via antidromic impulses, as ChR2-induced changes were preserved when lidocaine (4%, 200 nl) was injected in PB to block conduction at the soma. Each of the seven cells recorded with this approach (one OFF-cell, three ON-cells, three NEUTRAL-cells) responded to terminal activation both in baseline and during lidocaine block of PB cells bodies (*t*_6_ = 0.34, *p* = 0.74).

**Figure 7.**
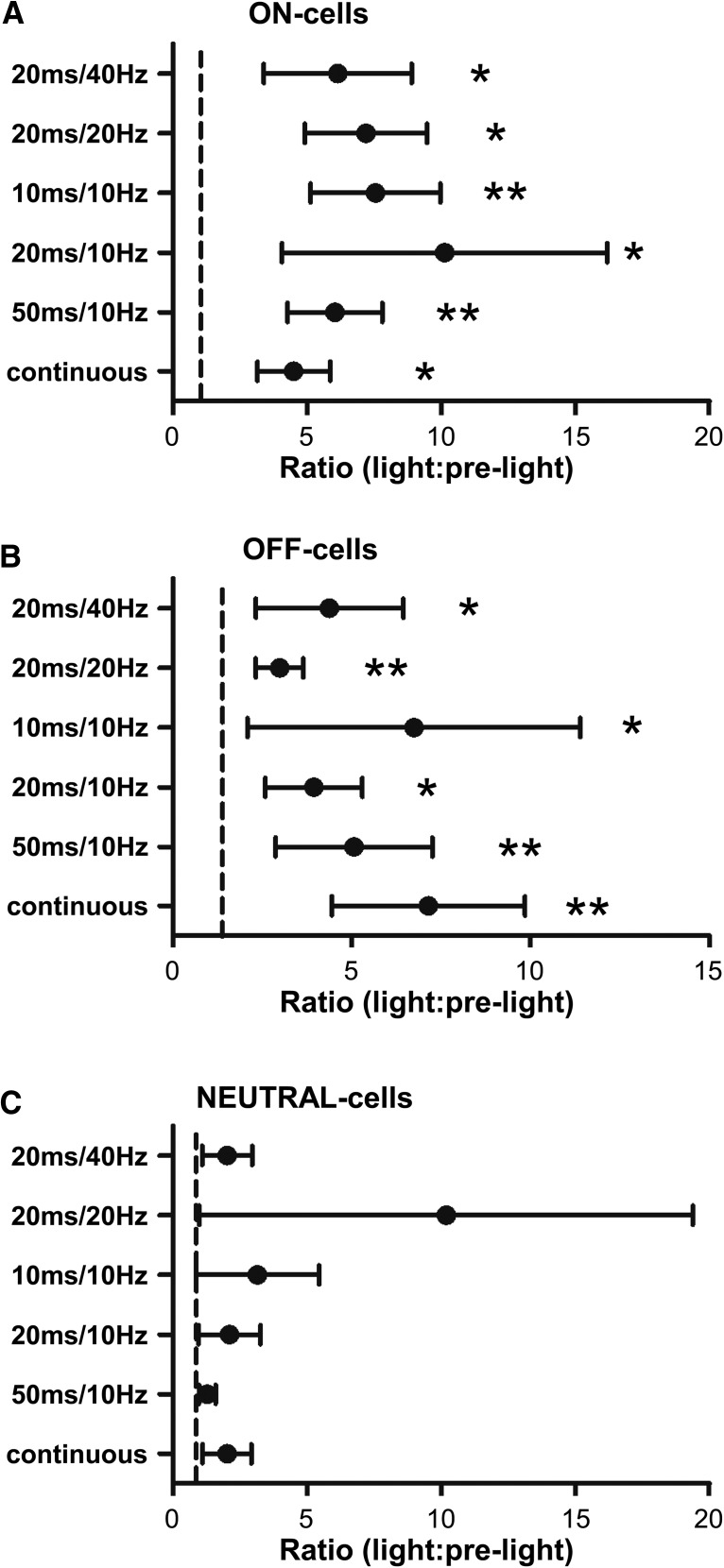
Effect of ChR2-induced activation of PB terminals on RVM neurons when using different stimulation protocols. As a class, ON-cells (***A***) and OFF-cells (***B***) showed significant and comparable responses to all light stimulation trains, whereas NEUTRAL-cells (***C***) did not respond during any protocol. Reported as geometric mean with 95% confidence intervals, *p < 0.05 and **p < 0.01 compared with the hypothetical value of 1 (no change), Wilcoxon’s signed rank test, *n* = 9–14 cells per class.

When comparing RVM neuronal activity during the light-on periods to the prelight periods, the different stimulation protocols tested were all capable of altering spontaneous firing of both ON- and OFF-cells, whereas they had no overall effect on NEUTRAL-cells. Net effects of terminal activation for different protocols are summarized in Figure 7 for each cell class. Individual neurons showed consistent responses across different stimulation protocols.

For those cells that exhibited an excitatory response, we determined the latency of the response to light-induced terminal activation. The OFF-cells responded at an average latency of 2.2 ± 0.2 ms from onset of the light pulse, consistent with a single synaptic delay. ON-cells responded at a significantly longer latency to activation of PB terminals, 3.5 ± 0.5 ms (unpaired *t* test, *t*_16_ = 2.54, *p* = 0.022, *n* = 18 cells). For the two NEUTRAL cells activated by terminal stimulation, the mean response latency was 2.1 ms (1.5 and 2.6 ms for the two cells). For the few cells that exhibited an inhibitory response to terminal activation, the response occurred at a much longer latency. For the two OFF-cells inhibited by light, the response occurred with an average delay of 139 ms, whereas that for the sole ON-cell was 228 ms, and for the NEUTRAL-cell was 161 ms. This long latency suggests that inhibition was an indirect response mediated by activity in local and/or remote circuits.

As might be expected given the increase in overall firing rate, the duration of the OFF-cell pause was reduced during activation of PB terminals (*t*_11_ = 6.88, *p* < 0.0001, *n* = 12; as noted above, firing of 2 of the 14 cells examined was suppressed during light stimulation, which precluded determination of a pause). The ON-cell burst was not changed significantly (*t*_12_ = 1.94, *p* = 0.076, *n* = 13).

Finally, consistent with the increase in overall OFF-cell firing, the heat-evoked paw withdrawal was delayed significantly with activation of PB terminals (latency_no light_ = 9.65 ± 0.18 s versus latency_light_ = 10.41 ± 0.27 s; W = −318.0, *n* = 28, *p* = 0.0003), indicating a modest anti-nociceptive effect of nonselectively activating PB input to RVM with these stimulus protocols.

Taken together, the *in vitro* and *in vivo* experiments using ChR2 to stimulate PB terminals in RVM demonstrate that PB terminals provide glutamatergic and GABAergic synaptic inputs to RVM neurons, and that direct inputs to the RVM can influence RVM pain-modulating neurons. The net effect of nonselective stimulation of PB input to RVM is predominantly excitatory. However, these experiments do not tell us what PB inputs to RVM are activated under any given physiologic or pathophysiological condition.

### Light-evoked inhibition of PB terminals in RVM using ArchT attenuates nociceptive response-related changes in RVM ON- and OFF-cell firing in vivo

To determine what PB inputs to RVM pain-modulating neurons are activated by acute noxious stimulation in an intact animal, we next used an optogenetic method to suppress activity in RVM-projecting PB terminals. In these experiments, we recorded RVM ON- and OFF-cell responses evoked by heat stimulation of the hindpaw in rats expressing ArchT in PB. We studied 15 ON-cells and 14 OFF-cells in 19 animals with light-induced inhibition of both PB terminals in RVM and PB cell bodies.

**Figure 8.**
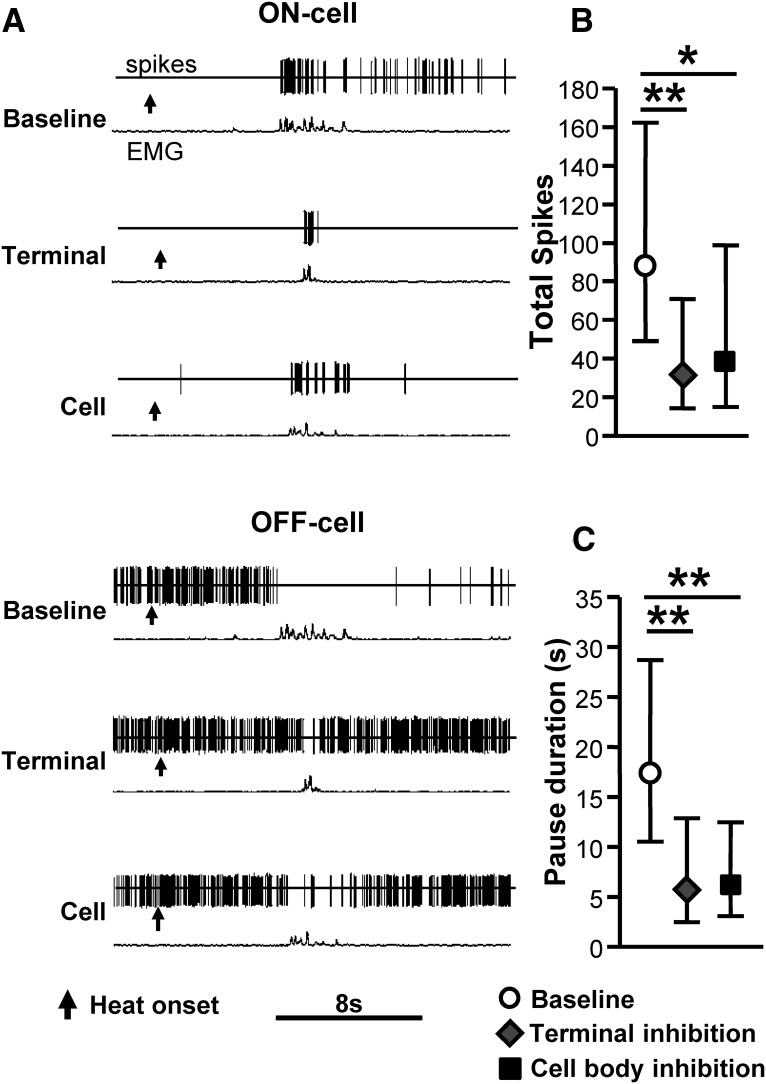
Effects of ArchT-induced inhibition of PB terminals and cell bodies on ON- and OFF-cell nociception-related activity. ***A***, Representative examples show ON- and OFF-cell activity during withdrawal from noxious heat stimulus at baseline compared with during Arch-T inhibition of PB terminals in RVM or at the cell bodies in PB itself. Arrow denotes heat onset. In both cases, nociception-related changes in firing were substantially attenuated. Summary data are provided in ***B***, ***C***. ***B***, ON-cells: effect of ArchT-induced inhibition of PB terminals and cell bodies on the ON-cell burst. ***C***, OFF-cells: the effect of ArchT-induced inhibition of PB terminals and cell bodies on the OFF-cell pause. Reported as geometric mean with 95% confidence intervals, *p < 0.05 and **p < 0.01 compared with baseline, one-way ANOVA with repeated measures and *post hoc* Dunn’s multiple comparisons test, *n* = 15 ON-cells, 14 OFF-cells.

Light-evoked inhibition of PB terminals in RVM significantly attenuated the ON-cell burst and OFF-cell pause (Fig. 8*A*), demonstrating that a direct projection from PB to the RVM conveys nociceptive information to RVM pain-modulating neurons. Light-evoked inhibition of cell bodies in PB also attenuated the ON-cell burst and OFF-cell pause evoked by noxious thermal stimulation, demonstrating that PB is a direct relay of nociceptive information to RVM ON- and OFF-cells. The effects of light-evoked suppression of PB terminals in RVM and cell bodies in PB are summarized for ON-cells (Fig. 8*B*) and OFF-cells (Fig. 8*C*).

**Figure 9.**
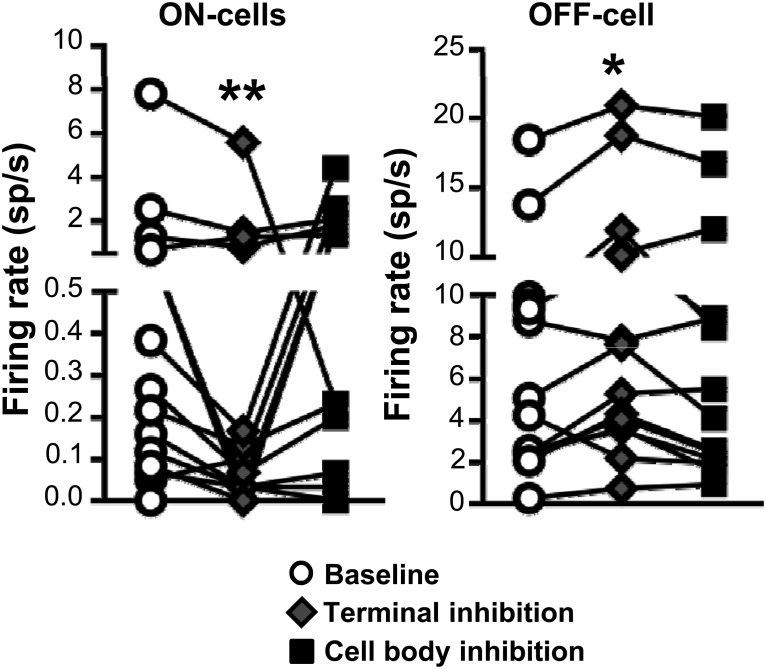
Effects of ArchT-induced inhibition of PB terminals and cell bodies on ongoing firing of ON- and OFF-cells in RVM. Terminal inhibition significantly decreased ON-cell ongoing firing, while increasing OFF-cell firing. Cell body inhibition did not significantly affect the ongoing firing of either ON- or OFF-cells. *p < 0.05 and **p < 0.01 compared with baseline, one-way ANOVA with repeated measures and *post hoc* Dunn’s multiple comparisons test, *n* = 15 ON-cells, 14 OFF-cells.

Light-evoked inhibition of activity in PB terminals in RVM also altered the ongoing discharges of both ON- and OFF-cells (Fig. 9), with a statistically significant decrease in ON-cell firing and increase in OFF-cell firing. However, the effect was quite modest (mean change of less than two spikes/s for both classes). Further, general inhibition of PB activity at the cell bodies did not produce significant changes in ON- and OFF-cell ongoing activity (Fig. 9). Any difference between the effects of terminal and cell body inhibition could be due to a failure to align the light probe with the densest expression of vector in PB itself (the terminal light probe was coupled to the recording site), inability to influence the full extent of the PB complex, or recruitment of long, extra-brainstem loops that have their own effects on RVM excitability. In any case, consistent with the minimal effect of terminal or cell body stimulation on the ongoing activity of ON- and OFF-cells, heat-evoked paw withdrawal latencies in animals with terminal or cell body inhibition were not significantly different from baseline (Friedman’s test, Fr = 3.79, *p* = 0.15, *n* = 19 animals; data not shown). However, the magnitude of the response was significantly reduced during inactivation of both terminals and cell bodies, to 64.4 ± 8.6% of no-light trials (*t*_18_ = 4.14, *p* = 0.0006 compared with 100%) and 58.3 ± 7.0% of no-light trials (*t*_18_ = 5.97, *p* < 0.0001), respectively.

## Discussion

### PB conveys nociceptive signals to RVM ON- and OFF-cells via a direct, intrabrainstem projection

Descending pain-modulating pathways regulate nociceptive processing via projections from the brainstem to the dorsal horn. The pronociceptive and anti-nociceptive outputs of RVM, mediated by ON- and OFF-cells, are themselves modulated by “top-down” inputs from areas including the amygdala, periaqueductal gray, and hypothalamus (Heinricher et al., 2009; Martenson et al., 2009; Heinricher and Fields, 2013). “Bottom-up,” nociceptive sensory inputs also play an important role in modulating RVM output: noxious stimuli activate pain-facilitating ON-cells, giving rise to a burst of firing, while suppressing the firing of pain-inhibiting OFF-cells, leading to a pause in any ongoing activity. These sudden changes in firing begin immediately before the behavioral withdrawal from the stimulus, allowing the response to occur by removing OFF-cell-mediated descending inhibition and enhancing the magnitude of the behavioral response (Fields and Heinricher, 1985; Jinks et al., 2007; Heinricher et al., 2010). Pain-modulating pathways are thus closely intertwined with pain-transmission pathways, forming a recurrent network that can only be fully understood from an integrative perspective.

PB is a nociceptive, emotional, and autonomic processing and relay center that, at least in rodent, is the target of the bulk of supraspinal projections from the superficial dorsal horn (Gauriau and Bernard, 2002; Todd, 2010). It also receives input from deep dorsal horn (Bernard et al., 1995; Bourgeais et al., 2001). PB was recently identified as an important relay of nociceptive information to the RVM (Roeder et al., 2016). However, that finding left open the question of whether there is a direct nociceptive connection from the PB to the RVM. PB could send nociceptive information to RVM indirectly, via structures such as the amygdala, insula, or periaqueductal gray, which receive input from PB and in turn project to RVM (Fulwiler and Saper, 1984; Jasmin et al., 1997; Gauriau and Bernard, 2002; Jasmin et al., 2004; Sato et al., 2013). Here we used optogenetic methods to determine if a direct projection from PB to RVM influenced activity of pain-modulating neurons and conveyed nociceptive information.

We used an inhibitory opsin, ArchT, to determine what PB inputs to RVM ON- and OFF-cells are activated by an acute noxious stimulus. In these experiments, optogenetic inactivation of PB terminals in the RVM substantially attenuated the nociceptive ON-cell burst and OFF-cell pause. This indicates that the direct projection from PB to RVM contributes to the ON-cell burst and OFF-cell pause. As with global inactivation of the PB area using the GABA_A_ receptor agonist muscimol (Roeder et al., 2016), neither the ON-cell burst nor the OFF-cell pause were completely eliminated with optogenetic inhibition of the PB terminals in RVM. This suggests that other inputs or relays also carry nociceptive information to the RVM. This could include direct spinal or trigeminal inputs to RVM, which have been documented in anatomic studies (Sugiyo et al., 2005).

### PB supplies both excitatory and inhibitory inputs to pain-modulating neurons in RVM

ArchT can be used to determine what inputs are activated by a given stimulus, as described above. By contrast, activation of PB terminals using ChR2 can provide evidence that certain inputs exist and have the potential to be activated, although it does not imply that they are in fact activated under any given physiologic or pathophysiological condition. ChR2-mediated activation of PB terminals in an RVM slice showed that PB conveys direct glutamatergic and GABAergic inputs to RVM neurons. The majority of responses evoked by PB terminal activation *in vitro* were glutamatergic (72%), with a smaller percentage of GABAergic inputs (28%). Furthermore, a given RVM neuron showed short-latency glutamatergic or GABAergic responses, but not both. This implies that direct PB synapses onto specific populations of RVM neurons are either glutamatergic or GABAergic. It is tempting to speculate that the GABAergic inputs revealed in the slice experiments map to the inhibitory nociceptive input to OFF-cells demonstrated *in vivo* using ArchT, since the inhibitory input responsible for the OFF-cell pause is known not to be mediated by local circuit interactions (Heinricher and McGaraughty, 1998; Cleary et al., 2008). It is also reasonable to suggest that at least some excitatory inputs identified in the slice were to ON-cells, since the *in vivo* experiments using ArchT confirmed an excitatory nociceptive input to neurons of this class. It is also possible that some of the neurons with glutamatergic inputs recorded in slice correspond to NEUTRAL-cells recorded *in vivo*.

The ArchT experiments showed that a net inhibitory input to OFF-cells and a net excitatory input to ON-cells from PB was recruited by noxious stimulation, yet the majority of ON- and OFF-cells surveyed were excited by ChR2 activation of PB terminals. These findings are not contradictory. As already noted, ArchT can be used to determine what inputs are activated by a given stimulus whereas activation of PB terminals using ChR2 provides evidence that certain inputs exist and have the potential to be activated. The excitatory responses to optogenetic activation of PB terminals occurred at short latency, consistent with a direct connection. These effects were not mediated by antidromic activation of PB cell bodies, as lidocaine injection in PB did not alter the light-induced responses of RVM neurons. These findings indicate that although PB relays a nociception-triggered inhibitory input to OFF-cells and excitatory input to ON-cells, additional direct projections from PB to RVM have the capacity to activate both classes of RVM pain-modulating neurons, ON- and OFF-cells, with the potential to be recruited under different conditions. These inputs, which would not have been predicted by a view of ON- and OFF-cells as receiving no information other than that driving the ON-cell burst and OFF-cell pause, presumably convey other influences to RVM pain-modulating neurons. For example, PB receives taste-related sensory-information (Sammons et al., 2016), which if relayed to the RVM could play a role in tastant-induced pain modulation (Anseloni et al., 2005; Foo and Mason, 2009). Different parabrachial circuits could also be recruited in persistent pain models. RVM neurons develop “sensitized” responses to normally non-noxious mechanical stimuli in neuropathic and inflammatory chronic pain models (Carlson et al., 2007; Cleary and Heinricher, 2013), and *in vitro* studies have shown that glutamatergic and GABAergic transmission, as well as effects of opioids, are altered in slices taken from CFA-treated animals (Guan et al., 2003; Guan et al., 2004; Zhang and Hammond, 2009; Zhang et al., 2013; Li et al., 2015, 2017). Delineating what PB inputs to RVM pain-modulating neurons are engaged under different conditions will require additional studies beyond the scope of the present manuscript.

The short latency of the OFF-cell activation by PB terminal activation *in vivo* is consistent with a single synaptic delay, which suggests that projections from PB form synaptic connections with RVM OFF-cells. The latency of the ON-cell response to PB terminal stimulation, although somewhat longer, is nonetheless consistent with a single synaptic delay. The difference in latencies between the two cell classes could be due to factors such as differential location of PB inputs on the ON-cells compared with the OFF-cells, or differences in intrinsic membrane properties. We also found a small percentage of ON- and OFF-cells that were inhibited by activation of local PB terminals. The latencies of these inhibitory responses were significantly longer than those of excitatory responses, implying that the short-latency inhibitory inputs demonstrated in the slice experiments and suggested by the ArchT experiments were masked by concurrent excitatory influences.

The ChR2 studies revealed a potential influence of PB on a subset of NEUTRAL-cells. Although the proportion of NEUTRAL-cells responding to activation of PB terminals in RVM was small, both activation and suppression of firing were seen. Since inactivation of PB terminals in the present studies and pharmacological block of PB itself had no effect on the firing of NEUTRAL-cells (Roeder et al., 2016), it would be interesting to know when and how parabrachial input to NEUTRAL-cells is engaged. It is likely that at least some of these NEUTRAL-cells serve other physiologic functions that are shared between PB and RVM, but unrelated to pain (e.g., thermogenesis; Morrison, 2011; Morrison et al., 2012).

### Implications for behavior

PB itself is not strongly implicated in acute nociceptive responding. Lesion of NK1-expressing neurons in dorsal horn, a major input to PB, has minimal (if any) effect on baseline nociceptive responding, although potentiated pain states are attenuated (Nichols et al., 1999; Suzuki et al., 2002; Rivat et al., 2009). Similarly, recruitment of RVM under extreme conditions can produce analgesia or hyperalgesia. For example, intense fear or pharmacological manipulations such as morphine eliminate the OFF-cell pause and produce analgesia. Conversely, sensitization of ON-cells and high levels of ongoing activity (10 Hz or more) contribute to hyperalgesia in inflammatory pain states. However, basal activity of RVM ON- and OFF-cell populations does not produce wide fluctuations in nociceptive responding, but instead contributes to the modest variations in nociceptive threshold that are more typical in daily life (Heinricher et al., 1989; Heinricher and Kaplan, 1991). Moreover, the ON-cell burst is not required for behavioral responses under baseline conditions, although it contributes to response magnitude (Heinricher and McGaraughty, 1998; Jinks et al., 2007). Conversely, reducing but not eliminating the OFF-cell pause does not produce potent analgesia (Heinricher et al., 2010).

In the present experiments, blocking PB terminals in RVM, which produced a slight shift in ON- and OFF-cell firing while substantially reducing the ON-cell burst and OFF-cell pause, had no effect on the latency of the heat-evoked paw withdrawal, but reduced the magnitude of the behavior. Conversely, the ChR2 stimulation protocol, which produced a moderate activation of both ON- or OFF-cells, resulted in a significant increase in response latency. Both outcomes are entirely what would have been expected given known effects of manipulating ON- and OFF-cell activity on acute nociceptive responding. Thus, the reduction in magnitude of the reflex with block of PB inputs to RVM was consistent with the reported reduction in response magnitude following selective block of the ON-cell burst (Jinks et al., 2007), since both the ON-cell burst and OFF-cell pause were decreased by inactivation of PB terminals. Conversely, the measurable increase in response latency during ChR2 activation of PB terminals is most likely explained by the measurable increase (<10 Hz) in overall OFF-cell firing. Studies in multiple persistent pain models will be needed to understand whether different PB inputs to RVM are engaged in different pain states, and will be an important next step in understanding this system.

### Conclusion

Although PB has been proposed as an important relay through which nociceptive information engages descending control circuitry important for diffuse noxious inhibitory controls (Lapirot et al., 2009), it was only recently that PB was established as a relay of nociceptive information to the RVM (Roeder et al., 2016). The present study demonstrates both excitatory and inhibitory synaptic input to RVM neurons from PB (ChR2 in RVM slice), and further shows that the direct projection from PB to RVM conveys acute nociceptive information to pain-modulating neurons in this region, with a net inhibitory input to OFF-cells and net excitatory input to ON-cells (ArchT *in vivo*). These observations are consistent with a circuit in which PB relays nociceptive input to suppress OFF-cell firing and enhance ON-cell firing.

The ArchT experiments demonstrated that PB provides net inhibitory input to OFF-cells during acute noxious stimulation and a net excitatory input to ON-cells. However, the ChR2 experiments revealed additional inputs beyond those activated by the acute noxious stimulus: excitatory influences on OFF-cells and inhibitory influences on ON-cells were also shown to exist. This raises the possibility that additional PB inputs to ON- and OFF-cells could be recruited under different conditions. Although RVM demonstrates significant, time-varying molecular, neurophysiological and functional plasticity in persistent pain states (Hurley and Hammond, 2000; Guan et al., 2004; Bie and Pan, 2007; Roberts et al., 2009; LaGraize et al., 2010; Leong et al., 2011), how these changes translate to enhanced pain remains poorly understood, in part because of the lack of an identified synapse that can be dissected in mechanistic studies. Given the importance of spinoparabrachial dorsal horn neurons in pathologic pain (Nichols et al., 1999; Suzuki et al., 2002), it is tempting to speculate that the inputs from PB to RVM identified here play a role in this well-documented plasticity. We suggest that the unexpected inputs revealed using ChR2 are activated or unmasked in persistent pain, and contribute to, or limit, expression of hyperalgesia. Isolating the synaptic mechanisms linking specific PB inputs to RVM ON- and OFF-cells could therefore provide unprecedented insights into the factors underlying sensitization of brainstem circuitry in persistent pain. The present studies thus both stand on their own in defining a significant pathway through which acute nociceptive information is conveyed to pain-modulating neurons of the RV and serve as a foundation for studies in persistent pain states.

## Synthesis

Reviewing Editor: Karen Davis, Krembil Research Institute and University of Toronto Decisions are customarily a result of the Reviewing Editor and the peer reviewers coming together and discussing their recommendations until a consensus is reached. When revisions are invited, a fact-based synthesis statement explaining their decision and outlining what is needed to prepare a revision will be listed below. Note: If this manuscript was transferred from JNeurosci and a decision was made to accept the manuscript without peer review, a brief statement to this effect will instead be what is listed below.

The authors have done a thorough job in responding to the previous reviews from the submission to J. Neuroscience and provide a convincing argument for the importance of the findings.
